# The interplay of forgiveness by God and self-forgiveness: a longitudinal study of moderating effects on stress overload in a religious Canadian sample

**DOI:** 10.1186/s40359-024-02238-y

**Published:** 2024-12-03

**Authors:** Sebastian Binyamin Skalski-Bednarz

**Affiliations:** 1 Faculty of Philosophy and Education, Katholische Universität Eichstätt-Ingolstadt, Luitpoldstraße 32, Eichstätt, 85071 Germany; 2 Institute of Psychology, Humanitas University, Sosnowiec, Poland

**Keywords:** Self-forgiveness, Forgiveness by God, Stress overload, Multilevel analysis, Religious Canadian sample

## Abstract

**Background:**

A consistent link between self-forgiveness and well-being has been established, yet a full understanding of self-forgiveness and its correlates, particularly in relation to forgiveness by God, remains limited, especially given that most existing data are cross-sectional. This study sought to address this gap by investigating the interplay between self-forgiveness and perceived forgiveness by God in reducing stress overload among religious individuals over time.

**Methods:**

This study involved 211 religious individuals in Canada, 55% of whom were female. Through multilevel analyses, the research examined the between-person, within-person, and cross-level effects of these forms of forgiveness on stress across three waves conducted over a total 12-month period.

**Results:**

The findings suggested that the effectiveness of self-forgiveness in mitigating stress may be significantly influenced by the perception of forgiveness by God, with the greatest stress reduction occurring when forgiveness by God was perceived at higher levels.

**Conclusions:**

These findings highlight the potential value of incorporating spiritual dimensions into psychological approaches to stress management, offering insights into the complex relationships between different forms of forgiveness and their impact on mental health of religious individuals. Future research is encouraged to further explore these dynamics across diverse cultural and religious contexts.

In the late twentieth century, the scientific exploration of forgiveness gained momentum, primarily focusing on interpersonal and self-forgiveness [1, 2]. However, despite the recognition of forgiveness as an important religious construct, forgiveness by God—deeply rooted in theological traditions—has been largely overlooked in empirical studies. This was partly due to a lack of differentiation between forgiveness by God and other forms of forgiveness. It was not until forgiveness by God was later identified as a separate dimension, with potentially unique psychological and spiritual effects, that researchers began to explore it [3, 4]. While this area has gained some academic attention in the early twenty-first century, its independent effects remain underexplored, and it continues to lag behind research on interpersonal and self-forgiveness [5–7].

## Forgiveness by God

Forgiveness by God, understood as forgiveness granted by the Supreme Being, is central to many world religions and plays a key role in the relationship between humans and a ‘higher power,’ providing a path to reconciliation [8, 9]. This process typically involves an individual's remorse or repentance, followed by forgiveness by God, which restores the relationship between the individual and this Higher Power [10]. Theological discussions often emphasize the readiness of God to forgive when repentance is sincere, as illustrated in religious narratives like King David's repentance in Christianity (Psalm 51:1) and Prophet Yunus's repentance in Islam (Qur'an 37:139–148).

The idea of unconditional forgiveness, where forgiveness by God might be granted without the offender’s repentance, is debated among theologians. Some have suggested that God's nature allows for forgiveness as an act of grace without preconditions [11]. Traditional views, however, maintain that forgiveness and reconciliation often require the wrongdoer's acknowledgment of guilt and a desire to change [10]. Forgiveness by God differs from related but closely intertwined concepts such as grace and mercy. While all involve unmerited favor, forgiveness by God specifically addresses an offense and often leads to a profound restoration of the relationship between the individual and the divine [12].

Research indicates that feeling forgiven by God plays a crucial role in alleviating negative emotions and psychological distress. Krause and Ellison [13] found that older adults who felt forgiven by God reported lower levels of depression and greater life satisfaction. Additionally, Lawler-Row [14] demonstrated that forgiveness by God can mediate the relationship between religiosity and health, linking it to positive emotional states like gratitude and inner peace.

Fincham [7] emphasized the importance of understanding forgiveness by God within a broader psychological and relational framework, integrating theological concepts with empirical research. His studies suggest that forgiveness by God is closely related to self-forgiveness and interpersonal forgiveness, indicating an interconnectedness that enhances well-being [6, 15]. However, the field remains fragmented, with research frequently constrained by study designs and an overemphasis on Christian populations in the United States, raising concerns about the generalizability of the findings [7, 15]. There is a need to further explore how forgiveness by God interacts with other forms of forgiveness, as studies suggest a cumulative effect on well-being [15, 16].

## Self-forgiveness

In exploring the relationship between forgiveness by God and self-forgiveness, the literature provides substantial evidence linking these concepts, though much of the focus has traditionally been on interpersonal forgiveness (e.g., [13, 14, 17, 18]). Self-forgiveness, which involves reducing self-condemnation for often the same transgressions for which believers seek forgiveness from God, is particularly intriguing. This process requires releasing negative emotions like guilt, shame, and self-condemnation while fostering compassion and self-acceptance [19–21].

The theoretical framework of self-forgiveness emphasizes reconciling conflicting self-identities: accepting responsibility for moral violations while also seeking self-acceptance [20, 22, 23]. This reconciliation prevents negative emotions from becoming pervasive and destructive, promoting psychological resilience. Self-forgiveness is not about excusing wrongdoing but integrating the experience into personal growth and moral development [24, 25]. Health benefits of self-forgiveness are well-documented, including lower levels of depression, anxiety, and stress, as well as higher levels of life satisfaction and well-being [26–29]. It reduces emotional burdens and facilitates healing, contributing to improved mental health outcomes [30–33].

Self-forgiveness also plays a crucial role in maintaining healthy interpersonal relationships, allowing individuals to move beyond mistakes, engage in reparative actions, and restore damaged relationships [34]. This relational aspect is particularly important in close relationships, enhancing empathy, understanding, and reconciliation [35]. Hall and Fincham [22] proposed that an individual's perception of forgiveness by God is closely linked to their ability to forgive themselves. Those who struggle with believing in or accepting forgiveness from a higher power may find it challenging to engage in self-forgiveness, which can hinder their spiritual and psychological healing. Cross-sectional studies have consistently shown a positive correlation between forgiveness by God and self-forgiveness [13, 36–39].

Research on the temporal dynamics of forgiveness has provided deeper insights into the relationship between self-forgiveness and forgiveness by God. One study found that changes in perceived forgiveness by God over 7 weeks were associated with self-forgiveness during that period [40]. Another study by Fincham et al. [6] revealed that perceived forgiveness by God predicted self-forgiveness 7 weeks later, even after accounting for initial levels of self-forgiveness. However, initial self-forgiveness did not influence later perceptions of forgiveness by God, suggesting that experiencing forgiveness by God may facilitate the process of self-forgiveness.

Moreover, Fincham and May [41] found that among young American adults, perceiving forgiveness by God moderated the relationship between self-forgiveness and psychological distress. The benefits of self-forgiveness in reducing depressive symptoms were most significant when levels of perceived forgiveness by God were high, while this effect was less pronounced when perceived forgiveness by God was low. Similarly, Skalski-Bednarz et al. [16] observed that higher self-forgiveness could mitigate the negative impact of stress on substance use cravings among Caribbean adults in Trinidad and Tobago, but this effect was only evident when perceived forgiveness by God was also high. These studies reported medium effect sizes, suggesting that while perceived forgiveness by God plays a moderating role in enhancing the positive effects of self-forgiveness on mental health, the strength of this relationship is moderate rather than overwhelming. Nevertheless, due to the cross-sectional nature of both studies, longitudinal research is needed to better assess the generalizability and establish the causal direction of these relationships.

## Current study

This study explored the relationship between self-forgiveness and perceived forgiveness by God in mitigating stress overload among religious individuals in Canada. Building on prior research primarily conducted with U.S. populations, we aimed to examine whether the psychological benefits of self-forgiveness and forgiveness by God observed in these studies would extend to the Canadian context. While Canada shares cultural similarities with the United States, its higher level of secularization and greater religious diversity may influence the relevance of religious coping resources, even in the context of religious individuals [42]. This study offers an opportunity to evaluate whether the significance of resources such as forgiveness by God remains consistent in a more secular environment, contributing to a better understanding of their psychological benefits across different cultural and religious contexts.

We employed multilevel analyses to examine the longitudinal associations between self-forgiveness, perceived forgiveness by God, and psychological distress, particularly in the context of stress overload—a prevalent issue in contemporary society. Specifically, our study investigated how self-forgiveness and perceived forgiveness by God interact to influence stress levels. We hypothesized that both self-forgiveness and perceived forgiveness by God would negatively predict experiences of general stress overload at the between-person, within-person, and cross-levels. Additionally, we proposed that perceived forgiveness by God would moderate the beneficial effects of self-forgiveness on reducing stress at these same levels, with the relationship being significant only when perceived forgiveness by God was high.

By employing a longitudinal design, this study addressed the limitations of cross-sectional research and attempted to provide more robust insights into the temporal dynamics of forgiveness and its effects on mental health. Data were collected at three time points over the course of a year, enabling a detailed analysis of how self-forgiveness and perceived forgiveness by God fluctuate over time and how these changes impact stress levels. This approach not only enhanced the generalizability of our findings but also offered a more nuanced understanding of the causal relationships between these variables.

## Materials and methods

We conducted this longitudinal study with a sample drawn from the general believer population across Canada, collecting data at three distinct intervals: May 2023, December 2023, and May 2024. The study was conducted in accordance with the approval of a university's ethics committee. Participants were recruited through the Prolific platform, which is particularly effective in targeting individuals with specific characteristics, such as religious affiliation or demographic profiles, ensuring that the sample closely matched the target population. Data collection was managed via Qualtrics. To be eligible for the study, participants were required to be of legal age, identify with a religious affiliation, and possess fluency in reading English. These eligibility criteria were verified before participants began the survey.

Participants who completed all three waves of the study were compensated with CAD $10. Invitations to participate in subsequent waves were delivered through Prolific. Importantly, at no point during data collection did the aggregated datasets contain any identifiable participant information. Each participant was assigned a unique code that remained consistent across all survey completions, ensuring anonymity while allowing for an accurate tracking of responses throughout the study. This approach upheld ethical research standards and safeguarded the privacy of all the participants involved.

### Sample size estimation

To ensure our study was adequately powered, we conducted an a priori sample size calculation. Using an anticipated medium effect size (Cohen's *f*2 = 0.25), a standard α level of 0.05, and a desired power of 0.8, we determined that a minimum sample size of 128 participants would be required for detecting main effects and 208 participants for detecting interaction effects. Our final sample size exceeded both thresholds, thereby providing sufficient power to detect meaningful effects with confidence.

### Participants

This study included 211 participants with a mean age of 39.2 (*SD* = 8.5) years, of whom 55% identified as female. In terms of race, 9% of participants identified as Black, while the remaining 91% were Caucasian; no other racial groups were reported. Participants' domiciles were categorized based on population size: 25% resided in small cities (population between 10,000 and 100,000), 35% in medium cities (population 100,000–1,000,000), 30% in large cities (population over 1,000,000), and 10% in rural areas (population under 10,000). The sample also reflected a diverse range of religious affiliations. Specifically, 49% identified as Protestant, 36% as Roman Catholic, 5% as Eastern Orthodox Christian, 8% as Muslim, and 2% as Hindu.

### Procedure

Across all three waves of the study, participants completed a series of questionnaires designed to evaluate their experiences of general stress overload over the past week, dispositional self-forgiveness, and their perception of forgiveness by God. Each assessment session was intentionally kept brief, taking approximately six minutes to complete. This streamlined approach was strategically employed to maximize participant retention, thereby improving the validity and relevance of the findings in relation to the proposed hypotheses.

### Measures

#### Experienced general stress overload

The Stress Overload Scale-Short Form (SOS-S) [43] was employed to assess the levels of general stress overload experienced by participants over the past week. This 10-item instrument evaluates two primary dimensions of stress: event load, which reflects the burden of external responsibilities and demands (e.g., "felt swamped by your responsibilities"), and personal vulnerability, which captures internal feelings of inadequacy and the perception that coping resources are insufficient (e.g., "felt like nothing was going right"). Participants rated their experiences on a 5-point response scale, ranging from 1 (*not at all*) to 5 (*a lot*). The item scores were aggregated, and mean scores were calculated to derive an overall measure of stress overload.

The SOS-S is well-regarded for its strong internal consistency and validity, making it a reliable tool for assessing stress across diverse populations [43]. In this study, we utilized only the overall stress index due to the strong intercorrelation between the subdimensions and the total score, as well as its high predictive validity for mental health disorders (in terms of sensitivity and specificity). The SOS-S demonstrated excellent reliability in our study, with Cronbach’s α values of 0.95 for the first wave, 0.94 for the second wave, and 0.95 for the third wave.

#### Self-forgiveness

The self-forgiveness subscale from the Forgiveness Scale (FS) [5] was employed to assess individuals' propensity to forgive themselves for past transgressions. This two-item subscale includes statements such as “I often feel that no matter what I do now, I will never make up for the mistakes I have made in the past” and “I find it hard to forgive myself for some of the things I have done wrong.” Participants responded on a 5-point scale, ranging from 1 (*strongly disagree*) to 5 (*strongly agree*). Validation studies have shown that the self-forgiveness subscale is positively associated with life satisfaction and negatively correlated with psychological distress, highlighting its effectiveness in exploring the psychological impacts of self-forgiveness [5].

In this study, the self-forgiveness subscale demonstrated acceptable internal consistency for a brief, two-item measure, with Cronbach’s α values of 0.61 in the first wave, 0.62 in the second wave, and 0.62 in the third wave. While these α values may appear lower than traditional thresholds, they are consistent with reliability expectations for short scales with fewer items, where internal consistency tends to be reduced due to limited content coverage [44]. Despite this, the subscale remains effective in capturing the psychological construct of self-forgiveness, with higher scores reflecting a greater propensity to forgive oneself.

#### Perceived forgiveness by God

The Divine Forgiveness Scale (DFS) [45], a four-item instrument, was utilized to measure the degree to which individuals perceived themselves as forgiven by God. Participants responded to statements such as “How often have you felt that God forgives you?” on a 5-point scale, ranging from 1 (*never*) to 5 (*many times*), and “I am certain that God forgives me when I seek His forgiveness,” on a 5-point scale, ranging from 1 (*strongly disagree*) to 5 (*strongly agre*e). Higher scores indicated a stronger perception of being forgiven by God. All religious affiliations declared by Canadian participants in the study use the concept of God to describe a Higher Power, making the use of this instrument appropriate for this sample.

Validation studies have demonstrated that the DFS is significantly associated with various aspects of religion/spirituality, as well as mental and physical health, supporting its utility in research exploring the intersections of faith and well-being [45]. In this study, the DFS demonstrated robust internal consistency, with Cronbach’s α values of 0.82 in the first wave, 0.82 in the second wave, and 0.84 in the third wave. Mean scores were reported in this analysis, with higher values indicating a greater perception of forgiveness by God. Although participants reported the frequency of situations in which they felt God had recently shown them mercy, the results should be interpreted as reflecting a general tendency, since the scale does not focus on a single, specific transgression but on the overall sense of forgiveness.

### Statistical analyses

All analyses were conducted using IBM’s SPSS Statistics (Version 29). Descriptive statistics, including means, standard deviations (*SD*s), skewness, and kurtosis, were calculated to evaluate the distribution of the variables. Correlations among controlled variables, multilevel correlations, and intraclass correlation coefficients (ICCs) were conducted to assess the relationships among the variables.

### Missing data

Data were collected online via a recruitment platform that required participants to respond to every item, resulting in a 72% retention rate across all three waves. Consequently, the dataset was considered complete, and no imputation of missing data was necessary.

### Multilevel regression analyses

To further evaluate these relationships, multilevel regression analyses were employed. Model fit was assessed using –2 log likelihood (–2LL), Akaike information criterion (AIC), and Bayesian information criterion (BIC). Lower values of −2LL, AIC, and BIC indicate a better fit of the model to the data, with AIC and BIC specifically balancing model fit with model complexity to avoid overfitting [46]. The α level for all statistical tests was set at 0.05.

A stepwise approach was used in the multilevel model analyses. Initially, an unconditional model of stress overload, including only the intercept, was constructed. Next, covariates such as age, sex, domicile, religious affiliation, and race were added to the model. In the third step, the main effects of self-forgiveness and perceived forgiveness by God were introduced. The fourth step incorporated interaction effects at both the between-person and within-person levels. Between-person interaction effects assessed whether the relationship between self-forgiveness and stress overload varied among individuals with differing perceptions of forgiveness by God. Within-person interaction effects examined whether changes in self-forgiveness influenced stress overload within individuals over time, depending on their momentary perceptions of forgiveness by God. Cross-level moderation effects were also included to investigate whether between-person perceptions of forgiveness by God moderated the within-person effects of self-forgiveness on stress overload. Covariates (i.e., age, sex, domicile, religious affiliation, race) and between-person predictors were grand-mean centered, while within-person predictors were person-mean centered, recommended when interaction terms are included at the within-person level, while grand-mean centering is used for interaction terms at the between-person level [47, 48]. In the final model, a random slope method was employed to test whether the within-person effects could be nested within the between-person effects [49].

### Interpretation of moderation effects

To facilitate the interpretation of moderation effects, scores on perceived forgiveness by God that were 1 *SD* above the mean were categorized as high perceived forgiveness by God, while scores 1 *SD* below the mean were categorized as low perceived forgiveness by God. Similarly, self-forgiveness scores were classified as high or low based on whether they were 1 *SD* above or below the mean, respectively.

## Results

Table 1 presents the descriptive statistics and correlations for the sample (*N* = 211). The mean levels of experienced general stress overload remained relatively stable across the three time (T) points (T1: *M* = 3.33, *SD* = 1.06; T2: *M* = 3.36, *SD* = 0.94; T3: *M* = 3.40, *SD* = 0.96). Skewness and kurtosis values indicated that all controlled variables were approximately normally distributed. Notably, experienced stress overload at T1 exhibited strong positive correlations with stress overload at both T2 (*r* = 0.74, *p* < 0.001) and T3 (*r* = 0.71, *p* < 0.001). Furthermore, self-forgiveness and perceived forgiveness by God were significantly correlated across time points, with both constructs showing significant negative correlations with experienced stress overload.

**Table 1:** Descriptive statistics and correlations for sample (*N* = 211)

Variable	*M* (*SD*)	*Sk*	*Kt*	1	2	3	4	5	6	7	8	9
1. Experienced General Stress Overload T1	3.33 (1.06)	–0.06	–0.99	–								
2. Experienced General Stress Overload T2	3.36 (0.94)	–0.22	–0.51	.74^***^	–							
3. Experienced General Stress Overload T3	3.4 (0.96)	–0.12	–0.47	.71^***^	.67^***^	–						
4. Self-Forgiveness T1	2.82 (1.96)	0.75	–0.79	–18^**^	–.19^**^	–18^**^	–					
5. Self-Forgiveness T2	2.99 (2.09)	0.49	–0.85	–.16^*^	–.2^**^	–.16^*^	.57^***^	–				
6. Self-Forgiveness T3	2.95 (1.96)	0.61	–0.89	–.15^*^	–.21^**^	–.15^*^	.54^***^	.56^***^	–			
7. Perceived Forgiveness by God T1	2.91 (1.57)	0.28	–0.98	–.45^***^	–.34^***^	–.22^***^	.23^***^	.19^**^	.23^***^	–		
8. Perceived Forgiveness by God T2	2.96 (1.33)	0.22	–0.87	–.38^***^	–.46^***^	–.23^***^	.2^**^	.22^**^	.2^**^	.71^***^	–	
9. Perceived Forgiveness by God T3	2.97 (1.36)	0.31	–0.92	–.38^***^	–.41^***^	–.21^**^	.17^*^	.18^**^	.18^**^	.69^***^	.74^**^	–
Age				–.19^**^	–.17^*^	–.21^**^	–.01	–.11	–.08	.17^*^	.15^*^	.15^*^
Sex (0 = male, 1 = female)				–.16^*^	–.17^*^	–.21^**^	–.01	.09	.08	.06	.05	.08
Race (0 = Caucasian, 1 = Black)				.17^*^	.19^**^	.18^**^	–.01	–.09	–.08	.21^**^	.22^**^	.22^**^

*Sk* Skewness*, Kt* Kurtosis, ^*^p < .05, ^**^p < .01, ^***^p < .001

Table 2 presents the multilevel correlations both between individuals and within individuals for the controlled variables. To further analyze the data, ICCs were calculated for the primary variables, providing insight into the proportion of variance attributable to differences between individuals. The ICC values were 43.2% for experienced general stress overload, 62.4% for self-forgiveness, and 66.7% for perceived forgiveness by God. These multilevel correlations indicated a considerable level of data dependency, validating the application of a multilevel modeling approach. Moreover, the results suggested ample within-person variance in both experienced general stress overload and self-forgiveness to warrant further analysis.

**Table 2:** Within-person and between-person correlations

Variable	Within-Person Relationships		Between-Person Relationships	
	1	2	1	2
1. Experienced General Stress Overload	–		–	
2. Self-Forgiveness	–0.15^*^	–	–0.17^*^	–
3. Perceived Forgiveness by God	–0.2^**^	0.18^**^	–0.49^***^	0.46^***^

^*^p < .05, ^**^p < .01, ^***^p < .001

Table 3 presents the five stages of multilevel modeling used to predict experienced general stress overload. The modeling process began with an unconditional model (Model 1), followed by the inclusion of between-person covariates (i.e., age, sex, domicile, religious affiliation, race) in Model 2 (pseudo-*R*^*2*^ = 0.08). Model 3 introduced the main effects of self-forgiveness and perceived forgiveness by God (pseudo-*R*^*2*^ = 0.21). Model 4 added the interaction effects at both the between-person and within-person levels (pseudo-*R*^*2*^ = 0.07). Finally, Model 5 incorporated the cross-level interaction effect (pseudo-*R*^*2*^ = 0.13). The reduction in the –2LL values across models, from Model 1 to Model 5, was statistically significant (all *p*s < 0.001), indicating a substantial improvement in model fit with each successive addition of predictors.

**Table 3:** Estimates of fixed and random effects from multilevel models predicting experienced general stress overload (*N* = 211)

Parameter Estimates (SE) – Unstandardized Coefficients					
	Model 1	Model 2	Model 3	Model 4	Model 5
Intercept	3.33^***^ (.07)	0.72^***^ (0.18)	1.21^***^ (0.19)	3.01^***^ (0.63)	1.47^***^ (0.22)
Female (Ref = Male)		–0.81^***^ (0.17)	–0.71^***^ (0.16)	–2.14^***^ (.0.39)	–0.77^***^ (0.16)
Black (Ref = Caucasian)		0.67^***^ (0.15)	0.49^***^ (0.14)	1.47^***^ (0.34)	0.54^***^ (0.14)
Small City Domicile (Ref = Rural)		0.34^***^ (0.05)	0.31^***^ (0.04)	0.78 (0.44)	0.28^*^ (0.14)
Medium City Domicile (Ref = Rural)		0.27^***^ (0.07)	0.21^***^ (0.06)	0.55^*^ (0.21)	0.18^**^ (0.06)
Large City Domicile (Ref = Rural)		0.48^***^ (0.11)	0.33^*^ (0.14)	0.95^***^ (0.14)	0.33^***^ (0.04)
Protestantism (Ref = Roman Catholicism)		0.16 (0.17)	–0.12 (0.17)	–0.23 (0.52)	–0.11 (0.17)
Eastern Orthodox Christianity (Ref = Roman Catholicism)		0.01 (0.11)	–0.13 (0.09)	–0.43 (0.31)	–0.18 (0.11)
Islam (Ref = Roman Catholicism)		0.05 (0.09)	–0.11 (0.09)	–0.42 (0.28)	–0.15 (0.09)
Hinduism (Ref = Roman Catholicism)		0.16 (0.23)	0.03 (0.22)	0.35 (0.66)	0.07 (0.21)
Age		–0.03^**^ (0.01)	–0.03^**^ (0.01)	–0.04^**^ (0.01)	–0.03^**^ (0.01)
BP Self-Forgiveness			–0.04 (0.03)	–0.12 (0.1)	–0.13 (0.11)
WP Self-Forgiveness			–0.02 (0.02)	–0.07 (0.07)	–0.03 (0.02)
BP Perceived Forgiveness by God			–0.12^***^ (0.02)	–0.42^***^ (0.05)	–0.22^***^ (0.04)
WP Perceived Forgiveness by God			–0.08^***^ (0.02)	–0.38^***^ (0.06)	–0.12^***^ (0.02)
BP Self-Forgiveness × Perceived Forgiveness by God				0.04^*^ (0.02)	0.02^*^ (0.01)
WP Self-Forgiveness × Perceived Forgiveness by God				0.06^*^ (0.03)	0.04^*^ (0.02)
**Random Effects**					
BP Residual Variance	0.07^***^ (0.01)	0.07^***^ (0.01)	0.05^***^ (0.01)	0.05^***^ (0.01)	0.06^***^ (0.01)
WP Residual Variance	0.09^***^ (0.01)	0.08^***^ (0.01)	0.07^***^ (0.01)	0.07^***^ (0.01)	0.06^***^ (0.01)
BP Perceived Forgiveness from God					–0.14^**^ (0.05)
**Fit indices**					
2LL	–5115.52	–4179.55	–3613.31	–3157.76	–2155.54
AIC	5135.04	4183.09	3156.62	3143.53	2141.08
BIC	5141.77	4123.48	3191.57	3190.65	2159.05
#Parameters	3	13	17	19	21

*BP* Between-Person Effect, *WP* Within-Person Effect, *AIC* Akaike Information Criterion, *BIC* Bayesian Information Criterion, *2LL* 2 × Log-Likelihood. Model Comparisons: Model 1 to Model 2: Δ2LL =  − 935.97, Δdf = 10, *p* < .001; Model 2 to Model 3: Δ2LL =  − 566.24, Δdf = 4, *p* < .001; Model 3 to Model 4: Δ2LL =  − 455.55, Δdf = 2, *p* < .001; Model 4 to Model 5: Δ2LL =  − 1002.22, Δdf = 2, *p* < .001

### Between-person effects

A significant main effect of perceived forgiveness by God on experienced general stress overload was identified at the between-person level (Model 3; see Table 3). Additionally, a significant interaction effect between self-forgiveness and perceived forgiveness by God on experienced general stress overload was observed, *b*_interaction_ = 0.04, *SE* = 0.02, *p* = 0.047. For individuals with higher levels of perceived forgiveness by God, self-forgiveness was significantly and negatively associated with experienced general stress overload (*p* = 0.013). However, for those with lower levels of perceived forgiveness by God, this association was nonsignificant (*p* = 0.584).

### Within-person effects

At the within-person level, significant main effects of perceived forgiveness by God on experienced general stress overload were also found (Model 3; see Table 3). Furthermore, a significant interaction between self-forgiveness and perceived forgiveness by God on experienced general stress overload was detected at the within-person level, *b*_interaction_ = 0.06, *SE* = 0.02, *p* = 0.003. Specifically, for individuals who perceived higher forgiveness by God, self-forgiveness was significantly and negatively associated with experienced general stress overload (*p* = 0.003). Conversely, for those who perceived lower forgiveness by God, this association was nonsignificant (*p* = 0.586).

### Cross-level effects

A significant cross-level interaction was identified where the between-person variable of perceived forgiveness by God moderated the within-person effects of self-forgiveness on experienced general stress overload, *b*_slope_ = –0.14, *SE* = 0.05, *p* = 0.006. Model 5, which incorporated this cross-level interaction effect, demonstrated the best fit to the data. Specifically, among individuals with higher levels of perceived forgiveness by God, self-forgiveness was significantly and negatively linked to experienced general stress overload (*p* = 0.01). Conversely, in individuals with lower perceived forgiveness by God, this relationship was nonsignificant (*p* = 0.592).

**Fig. 1:**
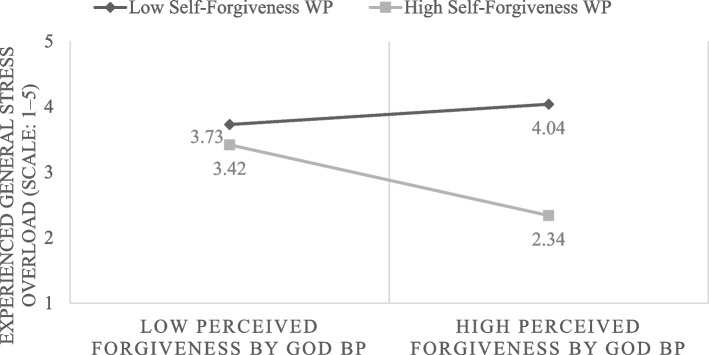
Interaction between the Within-Person (WP) effect of self-forgiveness and the Between-Person (BP) effect of perceived forgiveness by God in predicting general stress overload (*N* = 211)

To clearly visualize the identified interactions, Fig. 1 illustrates this cross-level interaction, which provided the best fit to the data. The figure illustrates how within-person self-forgiveness and between-person perceived forgiveness by God were jointly associated with stress overload. The plot reveals that among individuals with low perceived forgiveness by God, those with low self-forgiveness reported similar levels of stress overload as those with high self-forgiveness. However, individuals with both high perceived forgiveness by God and high self-forgiveness experienced notably lower stress overload compared to those with low self-forgiveness. Comparable patterns were observed in both the between-person and within-person interactions.

## Discussion

This study appears to be the first to utilize a multilevel approach to investigate the longitudinal associations between self-forgiveness, perceived forgiveness by God, and stress overload among religious individuals in Canada. The results confirmed that perceived forgiveness by God, both directly and in interaction with self-forgiveness, can predict reductions in stress overload at the between-person, within-person, and cross-levels over a 12-month period.

### Moderating role of perceived forgiveness by God

A key contribution of this study is the identification of the influential role of perceived forgiveness by God in shaping the relationship between self-forgiveness and stress overload. At both the between-person and within-person levels, perceived forgiveness by God moderated the impact of self-forgiveness on stress overload. This study further uncovered a significant cross-level interaction, wherein the between-person variable of perceived forgiveness by God moderated the within-person effects of self-forgiveness on stress overload. Specifically, individuals who engaged in self-forgiveness experienced a more consistent and pronounced reduction in stress overload when they also perceived higher levels of forgiveness by God. In contrast, no significant effect of self-forgiveness on stress was observed among those with lower levels of perceived forgiveness by God. This finding is consistent with prior research, which suggests that the interplay between self-forgiveness and forgiveness by God is crucial for psychological health [41].

Further supporting these findings, existing research has highlighted the protective role of forgiveness in mental health. For instance, perceiving forgiveness by God conditioned the mitigating effect of self-forgiveness on stress-related outcomes, such as substance use cravings, in Skalski-Bednarz et al. [16]. The importance of forgiveness by God as a moderator suggests that fostering its perception could enhance the effectiveness of self-forgiveness in managing stress among religious individuals, highlighting the need to integrate spiritual dimensions into psychological frameworks. This aligns with studies indicating that both self-forgiveness and forgiveness by God contribute to overall well-being, reducing depressive symptoms, enhancing resilience [5, 45, 50–52], and reinforcing the broader interplay between religious/spiritual resources and forgiveness [53–55].

Our findings suggest that the moderating effects of perceiving forgiveness by God are not limited to Trinidad and Tobago [16] and the United States [41], but are also evident in Canada. The consistent results across different countries and cultures indicate that the combined influence of self-forgiveness and perceived forgiveness by God on mental health may extend throughout North and South America. Moreover, although some religious groups were underrepresented in our sample, these effects were independent of religious affiliation, which did not emerge as a significant predictor in our analyses. Nevertheless, further research involving diverse cultural groups and populations is necessary to validate these preliminary conclusions.

### Main effect of perceived forgiveness by God

In addition to the interaction effects, the current study identified a main effect of perceived forgiveness by God observed at the between-person, within-person, and cross-levels. Notably, perceived forgiveness by God was found to predict stress behavior across these levels, with individuals who perceived higher levels of forgiveness by God consistently experiencing lower stress overload compared to those with lower levels of perceived forgiveness by God.

These results align with and build upon previous research, which has largely relied on cross-sectional data, by demonstrating that perceived forgiveness by God is a powerful factor in enhancing mental well-being ([13, 14, 17, 18]. Our study highlighted the significant role of perceived forgiveness by God in influencing stress management, both across different individuals and within the same individual over time, reflecting its dynamic nature.

Moreover, the within-person effects observed suggest that individuals’ perceptions of forgiveness by God can change in response to life circumstances, highlighting the fluid and transformative nature of perceived forgiveness by God, which is not a static characteristic but a dynamic factor that evolves over time. This evolving nature of forgiveness by God aligns with the insights of Goulding [56], who emphasized that God's mercy, which drives this forgiveness, is a process that can shape believers' perceptions and behaviors throughout their lives. Therefore, it is crucial to monitor how perceptions of forgiveness by God shift over time, rather than viewing them as fixed traits.

### Insignificant main effect of self-forgiveness

The absence of a main effect of self-forgiveness, despite a significant interaction effect, suggests that the positive influence of self-forgiveness on reducing stress within the context of general stress overload is largely dependent on the perception of being forgiven by God. Contrary to our hypothesis, self-forgiveness alone did not significantly reduce stress when the interaction effect was excluded from the model—an outcome observed consistently at the between-person, within-person, and cross-levels. This finding contrasts with some existing literature, such as Fincham and May [15], who demonstrated that interpersonal forgiveness, self-forgiveness, and forgiveness by God each uniquely contribute to mental health, underscoring their individual significance.

It is important to note, however, that Fincham and May's [15] study specifically focused on depressive symptoms, which differ from general stress management; elevated stress does not necessarily lead to depression. Self-forgiveness is often associated with feelings of shame, which are closely linked to negative self-esteem and can contribute to depressive affect and clinical depression [57]. In contrast, perceiving forgiveness by God fosters a connection with the divine, helping individuals cope with adversity by providing a sense of meaning—a coping mechanism distinct from the emotional processes associated with depression [7]. This suggests that different forms of forgiveness may serve varying functions depending on context and available psychological resources.

Recent research by Toussaint et al. [33] supports this perspective, showing that different forms of forgiveness can predict different health outcomes. In their clinical study, sensitivity to circumstances, unconditional forgiveness, and self-forgiveness positively predicted perceived health, while only forgiveness of others predicted life satisfaction. Notably, their study, conducted with a highly secular French sample, did not account for forgiveness by God.

Another possible explanation is that the perception of forgiveness from a supernatural entity (e.g., God) may vary depending on how individuals mentally represent that entity. Research (e.g., [58]) has identified two predominant views: a benevolent God (e.g., ‘forgiving,’ ‘loving’) and a wrathful God (e.g., ‘punishing,’ ‘stern’). Those who see God as benevolent may experience forgiveness more often but may attribute it to God's nature rather than their actions, thereby weakening the connection between self-forgiveness and stress reduction. Conversely, a restricted range of forgiveness experienced in those who see God as wrathful may heighten the importance of self-forgiveness in improving health outcomes.

Given that Canada is more secular than the United States, it is likely that cultural shifts have led to a reduced perception of a wrathful God in favor of a more benevolent and comforting one [42], which could influence the observed effects. Research suggests that individuals in more secularized societies are less inclined to perceive God as wrathful and are more likely to visualize God as a positive and benevolent figure [59]. This tendency is particularly pronounced in contexts where religious beliefs are less central to daily life, leading to a more abstract and less punitive concept of the divine [60]. This process of secularization may influence how forgiveness by God is perceived and, consequently, how it interacts with self-forgiveness in relation to stress and mental health. However, more research with Canadians is needed to evaluate a broader range of forgiveness dimensions beyond forgiveness by God and self-forgiveness in predicting various health outcomes, as well as to assess the mental representation of God as a potential moderator of these effects.

### Practical implications

The findings from this study offer valuable insights for mental health interventions, particularly those tailored to religious populations. Incorporating spiritual elements into therapeutic programs can significantly enhance their effectiveness, especially when the focus is on self-forgiveness. A practical example of this approach is the "Restore: The Journey Toward Self-Forgiveness" program, which has been shown to effectively foster self-forgiveness and improve mental well-being across various populations through structured writing exercises, reflective practices, and therapeutic activities [26, 32].

This integrated, spiritual intervention involves addressing an individual’s relationship with the divine and enhancing the individual’s perception of forgiveness by God. This approach can be particularly impactful in religious counseling or therapeutic settings where faith plays a central role in an individual’s life. Moreover, it can lead to not only improved mental health outcomes but also a strengthened sense of spiritual well-being [61, 62]. By combining self-forgiveness with perceived forgiveness by God, mental health interventions can provide a more holistic framework for reducing stress and promoting overall well-being.

Also, to enhance the perception of forgiveness by God, interventions might include guided meditations, prayer sessions, or discussions focused on understanding and internalizing divine mercy. These spiritual practices can be tailored to align with the individual’s beliefs and religious background, ensuring that the therapy resonates personally and maximizes its psychological benefits. By adopting these approaches, mental health professionals can more effectively meet the unique needs of religious individuals, potentially leading to better stress management and improved mental health outcomes. It is worth noting, however, that our findings primarily reflect the potential development of a positive orientation to better manage future adversities among healthy individuals in Canada, regardless of their religious affiliation (as no effect of religious affiliation was observed). Given the study’s limitations—such as the use of a non-clinical sample and the measurement of general forgiveness tendencies rather than forgiveness in response to a specific offense—the clinical relevance of these findings remains limited.

### Limitations and future directions

Despite its valuable contributions, this study had several limitations that warrant consideration. First, it was conducted with a general, non-clinical sample that did not constitute a representative sample of the Canadian population, and specific stressors or potentially traumatic experiences encountered by respondents were not controlled for. While the sample included individuals from various religious affiliations, some groups were underrepresented, reflecting the distribution of religious affiliations within Canada. As most participants identified as Christian, the applicability of these results to other religious groups remains uncertain. Future research should include more diverse populations to determine whether these relationships hold across different faith traditions and to ensure greater racial and ethnic diversity. Of particular interest would be further exploration of the concept of forgiveness by God within non-theistic religions such as Buddhism and Hinduism, given the limited data available in the existing literature. Additionally, understanding how self-forgiveness relates to stress overload and health among non-believers would be beneficial, as feeling forgiven by a Higher Power may play a unique role in fostering self-forgiveness among those who hold religious beliefs. For non-believers, alternative factors, such as personal alignment with widely accepted societal morals, could hypothetically serve as comparable moderators, potentially shaping the relationship between self-forgiveness and health or well-being outcomes [63, 64]. Notably, research on forgiveness interventions has shown that religiously oriented programs promoting forgiveness of others do not appear more effective than secular counterparts in improving overall well-being, except in their potential to enhance spiritual well-being [65].

Furthermore, while our longitudinal design provided valuable insights into the temporal dynamics of forgiveness and stress, the relatively short duration of 1 year may not have fully captured the long-term effects. Extending the follow-up period in future studies could offer a more nuanced understanding of the enduring impacts of these forgiveness processes.

Moreover, the reliance on self-report measures left the door open to potential biases that could have affected the accuracy of our findings. Although the use of self-report measures is supported by social-ecological theory—which posits that individuals' perceptions of their environment are more important in predicting behavior than objective reality [66]—relying solely on these measures often leads to common-method variance, inflating correlations among variables [67]. Incorporating more objective measures of stress, such as physiological indicators, alongside self-reported data, could enhance the validity of related future research.

## Conclusion

Overall, this study contributed to the literature by elucidating the complex, interdependent relationships between different forms of forgiveness and their collective impact on mental health. Through multilevel analysis that examined between-person, within-person, and cross-level effects, a significant interaction between self-forgiveness and forgiveness by God was identified among religious individuals in Canada. Our findings suggest that the effectiveness of self-forgiveness in reducing stress is strongly moderated by the perception of forgiveness by God, with the beneficial effects of self-forgiveness becoming pronounced only when forgiveness by God is perceived at higher levels. These results underscore the critical importance of integrating spiritual dimensions into psychological frameworks. Future research should continue to explore these dynamics across diverse cultural and religious contexts, utilizing longitudinal methodologies with extended durations to deepen our understanding of how forgiveness functions as a protective factor in stress management.

## Data Availability

The datasets used and analyzed during this study are available from the corresponding author upon reasonable request.

## Abbreviations

M: Mean
SD: Standard Deviation
BP: Between-Person
WP: Within-Person
ICC: Intraclass Correlation Coefficient
DFS: Divine Forgiveness Scale
SOS-S: Stress Overload Scale-Short Form
α: Cronbach's Alpha
AIC: Akaike Information Criterion
BIC: Bayesian Information Criterion
p: P-value (probability value)
